# Supplementary material for: Characterization of Novel Bacteriophage vB_KpnP_ZX1 and Its Depolymerases with Therapeutic Potential for K57 Klebsiella pneumoniae Infection
Source: Pharmaceutics. 2022 Sep 10;14(9):1916. doi: 10.3390/pharmaceutics14091916 (PMC9505181; doi:10.3390/pharmaceutics14091916)
Supplement: Supplementary file 1 [file pharmaceutics-14-01916-s001.zip › pharmaceutics-1889802-supplementary.pdf]

data

Supplementary Materials

Characterization of Novel Bacteriophage vB_KpnP_ZX1 and Its Depolymerases with Therapeutic Potential for K57 *Klebsiella pneumoniae* Infection

Ping Li [1,2,3], Wenjie Ma [1,2,3], Jiayin Shen [4,*] and Xin Zhou [1,2,3,*]

[1] College of Veterinary Medicine, Institute of Comparative Medicine, Yangzhou University, Yangzhou 225009, China
[2] Jiangsu Co-innovation Center for Prevention and Control of Important Animal Infectious Diseases and Zoonoses, Yangzhou University, Yangzhou 225009, China
[3] Joint International Research Laboratory of Agriculture and Agri-Product Safety, the Ministry of Education of China, Yangzhou University, Yangzhou 225009, China
[4] National Clinical Research Center for Infectious Diseases, The Third People's Hospital of Shenzhen, Shenzhen 518112, China
* Correspondence: johnnie1111@hotmail.com (J.S.); zhou_xin@yzu.edu.cn (X.Z.)

Abstract: A novel temperate phage vB_KpnP_ZX1 was isolated from hospital sewage samples using the clinically derived K57-type *Klebsiella pneumoniae* as a host. Phage vB_KpnP_ZX1, encoding three lysogen genes, the repressor, anti-repressor, and integrase, is the fourth phage of the genus *Uetakevirus*, family *Podoviridae*, ever discovered. Phage vB_KpnP_ZX1 did not show ideal bactericidal effect on *K. pneumoniae* 111-2, but TEM showed that the depolymerase Dep_ZX1 encoded on the short tail fiber protein has efficient capsule degradation activity. In vitro antibacterial results show that purified recombinant Dep_ZX1 can significantly prevent the formation of biofilm, degrade the formed biofilm, and improve the sensitivity of the bacteria in the biofilm to the antibiotics kanamycin, gentamicin, and streptomycin. Furthermore, the results of animal experiments show that 50 μg Dep_ZX1 can protect all *K. pneumoniae* 111-2-infected mice from death, whereas the control mice infected with the same dose of *K. pneumoniae* 111-2 all died. The degradation activity of Dep_ZX1 on capsular polysaccharide makes the bacteria weaken their resistance to immune cells, such as complement-mediated serum killing and phagocytosis, which are the key factors for its therapeutic action. In conclusion, Dep_ZX1 is a promising anti-virulence agent for the K57-type *K. pneumoniae* infection or biofilm diseases.

Keywords: *Klebsiella pneumoniae*; phage; depolymerase; capsule; biofilm; anti-virulence agent

1. Summary

Figure S1: string test of *K. pneumoniae* 111-2 (positive string test). Figure S2: the Western blots image of purified depolymerase Dep_ZX1. Table S1: primers used in this study, Table S2: antibiotic resistance of *K. pneumoniae* 111-2, and Table S3: open reading frames (ORFs) of phage vB_KpnP_ZX1.

2. Data Description

2.1. Figures and Tables

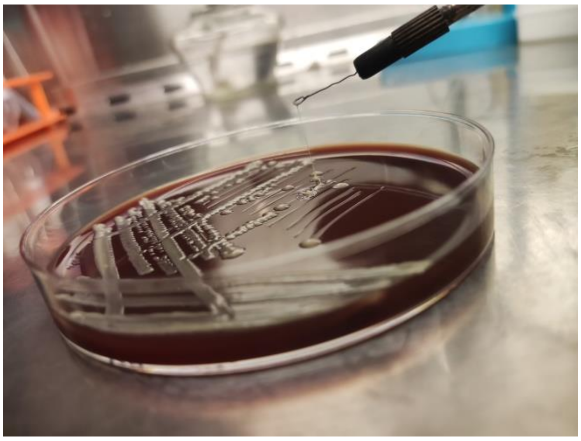

Figure S1. string test of *K. pneumoniae* 111-2 (positive string test). *K. pneumoniae* 111-2 colonies growing on blood plates can be pulled out by loops with sticky filaments ≥ 5 mm.

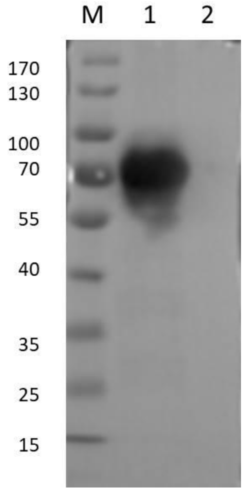

Figure S2. the Western blots image of purified depolymerase Dep_ZX1. 1: Purified recombinant Dep_ZX1; 2: Negative control, *E. coli* BL21 (DE3) carrying pET-28a.

Table S1. primers used in this study.

Primers	Sequence
Wzi-F	ATGATAAAAATTGCGCGC
Wzi-R	TAAACTAAACGGTATCTC
Wzc-F	GCATCGGTAACGAATAACAA
Wzc-R	TTTAGATTTATTATCTGT
RpmA-F	GCACATAAAAAGGCTGGC
RpmA-R	TTCAGCAACGATGCTGAT

Aerobactin-F	AAAAAGCGCCTCTGGGTG
Aerobactin-R	GAAGGTTAAGGCATAGTT
wabG-F	CGGACTGGCAGATCCATATC
wabG-R	ACCATCGGCCATTTGATAGA
allS-F	CACAAGACAACCCTGGAG
allS-R	GGGCGTCCGGAACAACGC
iucB-F	ATGTCTAAGGCAAACATCGT
iucB-R	TTACAGACCGACCTCCGTGA
urea-F	GAACTGACCCCCCGAGAA
urea-R	GATAATCGGGTTGTGAAC
fimH-F	CCTGTTCACCACCCTGCT
fimH-R	TGATAGACAAAGGTGATG
ybtA-F	GCGATCCGCTGGCGGAGG
ybtA-R	GTCAGAGCTGGCCGGGAG
mrKD-F	TCGCTGAGGAAATTACTA
mrKD-R	ATCGTACGTCAGGTTAAA
TFP-F	ATGACTGGTGGACAGCAAATGCAGGAGCTTGAAGGAAAA
TFP-R	CGCAAGCTTGTCGACGGAGCT CTTAATGTTGCCGTAATA

Table S2. antibiotic resistance of *K. pneumoniae* 111-2.

Antibiotic	Drug resistance
Piperacillin	S
Ampicillin	R
Ceftazidime	S
Cefuroxime	S
Cefoxitin	S
Cefatriaxone	S
Cefazolin	S
Cefotaxime	S

Imipenem	S
Amikacin	S
Gentamicin	I
Levofloxacin	S
Ciprofloxacin	S
Trimethoprim	S
Nitrofurantoin	S
Azithromycin	R
Erythromycin	R
Sulfamethoxazole	R
Tobramycin	I

S: sensitive, I: intermediate sensitive, R: resistant.

Table S3: Open reading frames (ORFs) of phage vB_KpnP_ZX1

ORFs	Start	Stop	ORF orientation (+–)	Length (bp)	Start codon	Function
ORF1	229	50	-	180	ATG	hypothetical protein
ORF2	291	524	+	234	ATG	hypothetical protein
ORF3	848	552	-	297	ATG	hypothetical protein
ORF4	1337	1194	-	144	ATG	hypothetical protein
ORF5	1711	1466	-	246	GTG	hypothetical protein
ORF6	2415	2224	-	192	GTG	hypothetical protein
ORF7	2734	2489	-	246	GTG	hypothetical protein
ORF8	3123	2773	-	351	ATG	hypothetical protein
ORF9	3332	3126	-	207	ATG	DNA-binding protein
ORF10	3589	3425	-	165	GTG	hypothetical protein
ORF11	4238	3630	-	609	ATG	hypothetical protein
ORF12	4603	4274	-	330	ATG	hypothetical protein
ORF13	5117	4581	-	537	ATG	hypothetical protein
ORF14	5359	5114	-	246	ATG	hypothetical protein
ORF15	5501	5370	-	132	ATG	hypothetical protein
ORF16	5677	5507	-	171	GTG	hypothetical protein

ORF17	6099	5686	-	414	ATG	regulator
ORF18	6908	6099	-	810	ATG	hypothetical protein
ORF19	7127	6918	-	210	ATG	glycosyltransferase
ORF20	7356	7117	-	240	ATG	hypothetical protein
ORF21	7880	7356	-	525	ATG	hypothetical protein
ORF22	8212	7889	-	324	ATG	hypothetical protein
ORF23	8910	8314	-	597	ATG	repressor
ORF24	9040	9324	+	285	ATG	transcriptional regulator
ORF25	9327	9635	+	309	ATG	RecA-dependent nuclease family protein
ORF26	9632	10066	+	435	ATG	endonuclease I-like superfamily protein
ORF27	10167	10514	+	348	GTG	hypothetical protein
ORF28	10566	11264	+	699	ATG	anti-repressor
ORF29	11686	12693	+	1008	GTG	site-specific DNA-methyltransferase HindIII
ORF30	12831	13136	+	306	ATG	hypothetical protein
ORF31	13133	13519	+	387	ATG	hypothetical protein
ORF32	13516	13668	+	153	ATG	hypothetical protein
ORF33	13901	14332	+	432	ATG	DUF551 domain-containing protein
ORF34	14332	14544	+	213	ATG	hypothetical protein
ORF35	14541	15359	+	819	ATG	hypothetical protein
ORF36	15406	15606	+	201	ATG	hypothetical protein
ORF37	15603	16067	+	465	ATG	ASCH domain-containing protein
ORF38	16064	17008	+	945	ATG	hypothetical protein
ORF39	17005	17757	+	753	ATG	hypothetical protein
ORF40	18014	17793	-	223	GTG	hypothetical protein
ORF41	18253	18011	-	243	ATG	pyruvate ferredoxin/flavodoxin

						oxidoreductase
ORF42	18516	18250	-	267	GTG	ANR family transcriptional regulator
ORF43	18715	18551	-	165	ATG	transcriptional regulator
ORF44	19051	18728	-	324	ATG	hypothetical protein
ORF45	19485	19219	-	267	ATG	hypothetical protein
ORF46	19574	20356	+	783	ATG	terminase small subunit
ORF47	20571	20353	-	219	ATG	transcriptional regulator
ORF48	20670	22289	+	1620	ATG	terminase large subunit
ORF49	22343	22774	+	432	ATG	hypothetical protein
ORF50	22767	25109	+	2343	GTG	portal protein
ORF51	25117	26037	+	921	ATG	hypothetical protein
ORF52	26049	26258	+	210	GTG	CsrA-like transcriptional regulator protein
ORF53	26357	27460	+	1104	ATG	major capsid protein
ORF54	27473	27937	+	465	ATG	hypothetical protein
ORF55	27989	28636	+	648	ATG	hypothetical protein
ORF56	28646	29296	+	651	ATG	hypothetical protein
ORF57	29296	30024	+	729	ATG	hypothetical protein
ORF58	30021	30638	+	618	ATG	hypothetical protein
ORF59	30604	33135	+	2532	GTG	endoglucanase
ORF60	33658	35502	+	1845	ATG	tail fiber protein
ORF61	35512	35796	+	285	ATG	hypothetical protein
ORF62	35891	36154	+	264	ATG	hypothetical protein
ORF63	36216	39095	+	2880	ATG	CalX-like domain protein
ORF64	39098	39463	+	366	ATG	tail fiber protein
ORF65	39460	41208	+	1749	ATG	hypothetical protein
ORF66	41208	41387	+	180	ATG	hypothetical protein
ORF67	41424	41879	+	456	ATG	GNAT family N-acetyltransferase
ORF68	41883	43133	+	1251	ATG	tail fiber domain-containing protein
ORF69	43133	43420	+	288	ATG	hypothetical protein
ORF70	43420	44601	+	1182	ATG	hypothetical protein
ORF71	44626	56229	+	11604	GTG	PLxRFG protein
ORF72	56483	57055	+	573	ATG	endolysin
ORF73	57055	57405	+	351	ATG	holin
ORF74	57402	57731	+	330	ATG	hypothetical protein
ORF75	57728	58294	+	567	ATG	Rz lysis protein
ORF76	59176	59412	+	237	ATG	hypothetical protein
ORF77	60705	59440	-	1266	ATG	Integrase

ORF78	60981	60724	-	258	GTG	hypothetical protein

3. Methods

The string test of bacteria is to pick up a single colony of *K. pneumoniae* 111-2 cultured overnight on the blood agar plate with a sterile inoculation ring. The positive hypermucoviscous phenotype is that bacteria can form strings≥5 mm.

The purified depolymerase Dep_ZX1 was characterized by Western blotting. The samples were transferred to PVDF transfer membrane (Merck KGaA, Darmstadt, Germany) after SDS-PAGE. Then samples were incubated with 5% PBSA for 2 h, anti-His-Tag mouse monoclonal antibody (Cwbio, Taizhou, China) for 2 h, and goat anti-mouse IgG conjugated to horse-radish peroxidase (Cwbio) for 1 h. The immunoreactive protein bands were observed by electrochemiluminescence detection system (Tanon, shanghai, China).

Antibiotic resistance of bacteria was determined by standard Kirby-Bauer disk diffusion method of Clinical Laboratory Standard Institute.

The Open reading frames (ORFs) of phage vB_KpnP_ZX1 were predicted by RAST and alignment by blastX in NCBI.
